# Degradation of Neonicotinoids and Caffeine from Surface Water by Photolysis

**DOI:** 10.3390/molecules26237277

**Published:** 2021-11-30

**Authors:** Alexandra Raschitor, Alberto Romero, Sandra Sanches, Vanessa J. Pereira, Joao G. Crespo, Javier Llanos

**Affiliations:** 1 Chemical Engineering Department, University of Castilla-La Mancha, Edificio Enrique Costa Novella, Campus Universitario s/n, 13005 Ciudad Real, Spain; alexandra_raschitor@yahoo.com (A.R.); alberto.romero7@alu.uclm.es (A.R.); 2 iBET—Instituto de Biologia Experimental e Tecnológica, Apartado 12, 2780-901 Oeiras, Portugal; sandramsanches@gmail.com; 3 Instituto de Tecnologia Química e Biológica António Xavier, Universidade Nova de Lisboa, Av. da República, 2780-157 Oeiras, Portugal; 4 LAQV-REQUIMTE, Department of Chemistry, NOVA School of Science and Technology, FCT NOVA, Universidade NOVA de Lisboa, 2829-516 Caparica, Portugal; jgc@fct.unl.pt

**Keywords:** neonicotinoids, caffeine, drinking water treatment, direct photolysis, advanced oxidation process, ecotoxicity

## Abstract

Along with rapid social development, the use of insecticides and caffeine-containing products increases, a trend that is also reflected in the composition of surface waters. This study is focused on the phototreatment of a surface water containing three neonicotinoids (imidacloprid, thiamethoxam, and clothianidin) and caffeine. Firstly, the radiation absorption of the target pollutants and the effect of the water matrix components were evaluated. It was observed that the maximum absorption peaks appear at wavelengths ranging from 246 to 274 nm, and that the water matrix did not affect the efficiency of the removal of the target pollutants. It was found that the insecticides were efficiently removed after a very short exposure to UV irradiation, while the addition of hydrogen peroxide was needed for an efficient caffeine depletion. The electrical energy per order was estimated, being the lowest energy required (9.5 kWh m^−3^ order^−1^) for the depletion of thiamethoxan by indirect photolysis, and a concentration of hydrogen peroxide of 5 mg dm^−3^. Finally, a preliminary evaluation on the formation of by-products reveals that these compounds play a key role in the evolution of the ecotoxicity of the samples, and that the application of direct photolysis reduces the concentration of these intermediates.

## 1. Introduction

Neonicotinoids are a class of insecticides that includes imidacloprid, acetamiprid, thiacloprid, dinotefuran, nitenpyram, thiamethoxam, and clothianidin. This class represents about 20% of the actual global insecticide market. Due to their high target specificity to insects, and their relatively low risk for non-targeted mammalian species, these insecticides are commonly used in agriculture and veterinary medicine, mostly for cutaneous applications [1,2,3,4]. The neonicotinoids act on postsynaptic nicotinic acetylcholine receptors. In insects, these receptors are located entirely in the central nervous system, inducing paralysis and death. According to Bonmatin et al. [5], neonicotinoids are expected to accumulate in soils, since their half-lives can exceed 1000 days. Even though these compounds were reported in laboratory studies to be susceptible to rapid degradation through photolysis (e.g., aqueous photolysis DT50 < 1 day for example for clothianidin), slow rates of dissipation have been described under field conditions. The authors state that the high-to-moderate solubility, leaching potential, and persistence of most neonicotinoids cause a continuing and increasing risk to the aqueous environment [5].

Since 2015, neonicotinoid insecticides have been added to the watchlist of substances that could potentially represent a threat for the environment in an European Union monitoring program (495/2015/EU) [6,7]. This document was further modified in 2018 to 840/2018 EU 7. The objective of this list, which contains 17 compounds, is to collect high-quality monitoring data and to support their prioritization. 

Caffeine, on the other hand, is the most highly consumed psychoactive drug, which is found, apart from coffee, in some teas, energy drinks, sodas, and chocolate. Its presence in water bodies was found to be related to human agglomerations. Higher levels were found in cities; meanwhile, traces were found in remote villages [8,9,10]. Moreover, caffeine concentrations were observed to increase closer to wastewater treatment plants and exhibited laboratory-scale apparent half-lives, ranging from 5 to 23 h in different matrices [11].

The neonicotinoid insecticides imidacloprid, clothianidin, and thiamethoxam, and the psychoactive stimulant caffeine, have been detected worldwide in surface waters [12,13,14,15].

Sadaria et al. [16] conducted the first nationwide wastewater reconnaissance study in the United States that was focused on the occurrence of neonicotinoid insecticides. Based on the results obtained in 13 conventional wastewater treatment plants, the authors extrapolate that annual discharges, of the order of 1000−3400 kg/y of imidacloprid, can be expected in the treated effluents nationwide and released to surface waters. The authors identified imidacloprid, acetamiprid, and clothianidin as recalcitrant sewage constituents that persist through 13 different conventional wastewater treatment facilities to enter water bodies at significant loadings, potentially harmful to sensitive aquatic invertebrates.

The simultaneous presence of various pollutants, such as complex mixtures, and a long-term exposure can lead to serious chronic effects, as seen in several studies [17,18]. Their effects can gradually accumulate, leading to irreversible changes on both wildlife and anthropogenic ecosystems [19].

Direct and indirect photolysis are promising treatment processes due to their effectiveness for deactivating pathogens and degrading chemical contaminants, their low cost, and their wide range of applications [20,21]. Several works have studied the degradation of imidacloprid (an insect neurotoxin that belongs to the neonicotinoids group) using photolysis. Among them, Englert et al. studied the influence of UV-A and UV-B irradiation for decreasing the toxicity of this compound coming from leaves falling from treated trees [22], and Zhang et al. evaluated the effect of the presence of dissolved organic matter in the degradation of imidacloprid, observing an increase in degradation for increasing organic matter concentrations [23]. Additional works have also evaluated the efficiency of Ag-ZnO composites in the heterogeneous photocatalytic degradation of imidacloprid [24]. All these works focused on the degradation of a single neonicotinoid and studied only one or two of the following topics—the degradation kinetics, the detection of intermediates, or the evolution of toxicity—but did not cover all these aspects of the treatment in a comprehensive study. Recently, an additional paper studied the degradation of a single neonicotinoid (acetamiprid), covering both the analysis of degradation products and their toxicity [25].

Other works have focused on the degradation of mixtures of neonicotinoids by solar irradiation, and have covered important topics, such as the detection of reaction intermediates [26] or the calculation of their quantum yields [27]. Direct photolysis, using low-pressure mercury lamps that emit monochromatic light at 254 nm, and indirect photolysis has been tested and proposed as a treatment option for degrading neonicotinoids [28,29,30].

Those latter works concluded that the use of heterogeneous photocatalysis, or the addition of hydrogen peroxide, improves the performance of the degradation process, but they did not perform a toxicity analysis to address the generation of intermediates. Medium-pressure mercury lamps should be tested as an alternative to low-pressure mercury lamps for the treatment of these pollutants, since for a compound to be degraded by direct photolysis, it needs to be able to absorb the light emitted by the lamps. Lamps that emit polychromatic light may, thus, prove to be more effective for achieving an effective degradation without the need to add chemical compounds. 

Additionally, several works have reported the treatment of caffeine by photolysis, which has been classified in the group of most-refractory emerging pollutants to the treatment by low-pressure and medium-pressure UV irradiation [31]. In this field, the activation of oxidants, such as peroxymonosulfate [32] or carbonate radicals [33], in the degradation of caffeine by photolysis has also been studied, concluding that the water matrix may play an important role in the degradation of this refractory pollutant.

To the best of the authors’ knowledge, there is not a previous comprehensive study that covers the degradation of mixtures of neonicotinoids and caffeine (two groups of chemicals with a very dissimilar behaviour) in real water using a medium-pressure mercury lamp and advanced oxidation processes by the addition of hydrogen peroxide, and that evaluates the toxicity of the treated effluent.

Based on this, the present study is focused on the treatment of real surface water spiked with a mixture of common pollutants, such as three neonicotinoids insecticides (imidacloprid, clothianidin, and thiamethoxam) and caffeine. Direct and indirect photolysis are compared, observing the influence of the hydrogen peroxide dosage, and determining the global efficiency of the process by performing an ecotoxicity analysis and a preliminary evaluation of the formation of by-products.

## 2. Materials and Methods

### 2.1. Reagents

The insecticides (imidacloprid, clothianidin, and thiamethoxam) and caffeine used in this study were purchased as solids of the highest commercially available grade (Sigma-Aldrich, Darmstadt, Germany). The aqueous suspension of bovine liver catalase with 40,000–60,000 units mg^−1^ was also obtained from Sigma-Aldrich; meanwhile, hydrogen peroxide (30% *w*/*w*) was obtained from Carlo Erba (Sabadell, Spain). 

Concentrations of 500 μg/L of each pollutant were spiked in real surface water. This concentration was defined to carry out all the experiments described in this paper, to ensure that the target compounds would be well above the detection limits of direct injection HPLC (5 µg/L) so that degradation could be monitored. Two water matrices were compared: deionized water (DW) and real surface water (SW) collected from the Tagus river (Lisbon, Portugal) and filtered with a 0.45 µm membrane filter (PALL, NY, USA). Although the composition of the raw water changes with time, the average dissolved organic carbon value of the filtered water was 2.35 mg/L, the pH was 7.64, and the turbidity was 0.22 NTU [34].

### 2.2. Experimental

The photolysis experiments were carried out in a collimated beam bench-scale reactor. This reactor was equipped with a polychromatic medium-pressure lamp, UVH type Z (UV-Technik, Meyer gmbh, Ortenberg, Bleichenbach, DE), housed in a shuttered box with PN310 quartz (UV-Technik, DE). The medium-pressure UV lamps offer a broad, pronounced line spectrum in the ultraviolet and visible range (200 nm to 600 nm) and a high-power density of about 100 W/cm^2^. For disinfection and degradation to occur by direct photolysis (the direct action of UV light), the microorganisms and chemical compounds to be treated need to have the capacity to absorb UV light. Low-pressure mercury lamps that emit monochromatic light are often applied for the disinfection of different water pathogens due to the light-absorption of DNA. Medium-pressure mercury lamps that emit polychromatic light in a wide range of wavelengths are, therefore, more effective for achieving not only disinfection but also the degradation of photolabile chemical compounds that have the capacity to absorb the emitted UV light. 

The samples were analyzed by high-performance liquid chromatography equipped with a photodiode array detector (Waters Chromatography, Milford, CT, USA). A column-type Luna, 5 µm C18, 100 A (150 × 3 mm) (Phenomenex Inc., Torrance, CA, USA, EE. UU.), was used. The isocratic mobile phase composition used was 0.1 % (*v*/*v*) formic acid in laboratory-grade water (85%) and acetonitrile (15%), with a flowrate of 0.6 mL/min and a column temperature of 40 °C. The wavelengths monitored by the photodiode array detector for the detection of the target compounds were 251 nm (thiamethoxam), 268 nm (clothianidin and imidacloprid), and 272 nm (caffeine). 

In the indirect photolysis tests, concentrations between 5 and 60 mg/L of hydrogen peroxide were added at the beginning of the assay, and bovine liver catalase was added to each sample at its collecting moment in order to stop the further reaction of OH⦁ radicals with undegraded organic matter.

The electrical energy per order (EEO, kWh m^−3^ order^−1^), which is the energy required for the degradation of 90% of the pollutant, was calculated using Equation (1) [35]:

(1) $$E_{EO}\;=\;\frac{6.39\cdot 10^{-4}\cdot P}{V\cdot K}$$

where 6.39 × 10^−4^ is a conversion factor (ln10 × 1 h/3600 s), *P* is the electric power of the lamp (2500 W), *V* the volume of the cell (0.05 L), and *K* is the kinetic constant, assuming first-order kinetics (s^−1^).

The ecotoxicity test was performed by evaluating the mobility of the microcrustaceous *Daphnia magna* using the Daphtoxkit F magna (ISO 6341) [36]. The *Daphnia magna* culture was prepared two days before starting the test. All the content of a vial containing the culture was transferred into a microsieve, and after subsequent rinsing with Mili-Q water, the *Daphnia magna* larvae were transferred to a petri dish large enough to store all the crustaceans and the nutrients used. In order to avoid death from the starving of the culture, pre-feeding with a solution containing *Spirulina* was added 2 h before placing the samples in contact with the culture in the test plates. After the crustaceans were transferred into each sample, dilutions (1:10 and 1:100) were prepared in the standard freshwater supplied in the kit, and data concerning the loss of mobility was taken after 24 and 48 h of incubation at 20 ± 2 °C in the dark.

## 3. Results

### 3.1. Removal of a Mixture of Neonicotinoids and Caffeine by Direct Photolysis

**Figure 1:**
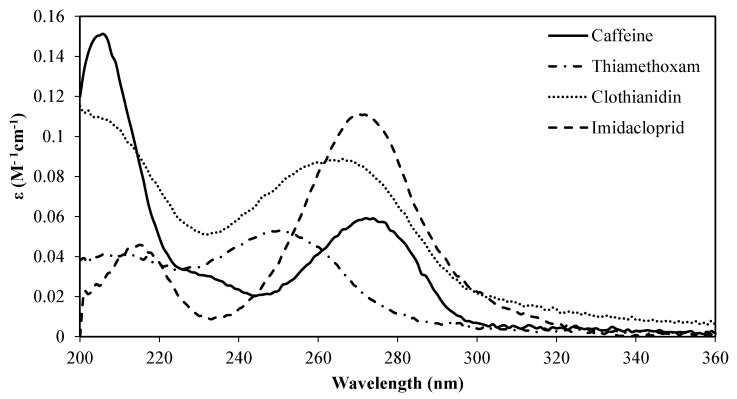
Decadic molar extinction coefficient of the pollutants.

Figure 1 shows the decadic molar extinction coefficient of the pollutants present in the laboratory-grade water spiked with the compounds under study at room temperature. This analysis plays a very important role in understanding whether the light emitted by the lamps used can be absorbed by the target pollutants. 

As can be seen, caffeine absorbs at wavelengths between 243 and 302 nm, reaching, in this range, a maximum decadic molar extinction coefficient of 0.059 M^−1^cm^−1^ at 274 nm. Regarding the insecticides, it can be observed that the imidacloprid and clothianidin show a maximum decadic molar extinction coefficient at a wavelength close to caffeine—of 0.11 M^−1^cm^−1^ at 272 nm for imidacloprid, and 0.088 M^−1^cm^−1^ at 264 nm for clothianidin—while thiamethoxam shows its maximum decadic molar extinction coefficient of 0.053 M^−1^cm^−1^ at 251 nm. To be degraded by direct photolysis, a compound must have the capacity to absorb the emitted light. These results, thus, show that the target pollutants are able to absorb the light emitted by the medium-pressure mercury lamp used in this study, and that medium-pressure mercury lamps that emit polychromatic light are expected to be more effective for degrading the target pollutants than low-pressure mercury lamps that emit monochromatic light at 254 nm. 

**Figure 2:**
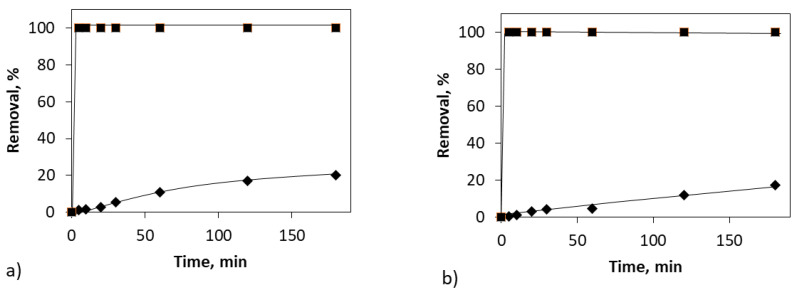
Influence of the water matrix on the pollutant removal by direct photolysis. (**a**) DW; (**b**) SW. ♦—caffeine; ■—insecticides mix.

In Figure 2, it can be observed the removal percentage of the insecticide mixture and caffeine in direct photolysis assays for two different water matrices: DW and SW. Due to the similar removal of the three insecticides at the sampling times of these tests, only one series, regarding the removal of the insecticides mix, was used in this plot. As can be seen, UV irradiation is capable of removing the insecticides to levels below detection by direct injection (5 μg/L), regardless of the water matrix, in a very short time. Unfortunately, caffeine behaves as a much more refractory compound to be degraded by UV irradiation. Although the removal of caffeine seems to be slightly faster for the DW water matrix, the removal rate for caffeine is far below that registered for the mix of insecticides for both systems. 

Due to the fast removal of the insecticides, a new assay was proposed by taking samples in a shorter time interval, in order to observe better the kinetics of their treatment. Taking into account that the water matrix does not seem to appreciably affect the removal of the pollutants, from that point on, the tests were carried out using SW as matrix. 

**Figure 3:**
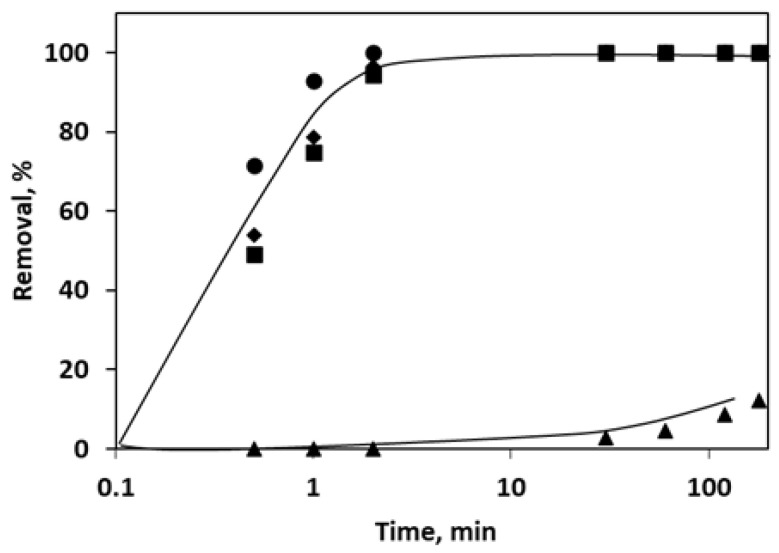
Pollutant removal using SW: ▲—caffeine; ■—imidacloprid; ●—clothianidin; ♦—thiamethoxam.

Figure 3 shows the removal degree of the pollutants at shorter sampling times, represented in semilogarithmic scale. The last sample was taken at 180 min.

As can be seen, the insecticides are efficiently removed in the first two minutes of irradiation exposure, while caffeine begins to degrade only after 50 min of exposure and reaches a maximum of 15% after 3 h of treatment. Another interesting result that can be observed is that even if the three insecticides have very similar structures, clothianidin is the first to be depleted, after only 1 min of exposure.

Many studies have shown the efficiency of depleting organic compounds using direct photolysis without the need of adding other chemicals. However, the times that are needed to achieve effective degradation are normally much higher than what was observed for the insecticides. As mentioned before, in the first s nationwide wastewater reconnaissance study in the United States that was focused on the occurrence of neonicotinoid insecticides, Sadaria et al. [16] identified imidacloprid, acetamiprid, and clothianidin as recalcitrant sewage constituents that persist through 13 different conventional wastewater treatment facilities. Three of the tested wastewater utilities performed UV disinfection instead of chlorination. Since UV disinfection is mentioned in the manuscript [16], we assume the most commonly used lamps for disinfection were used, which consist of low-pressure UV mercury lamps that emit monochromatic light at 254 nm and that are widely applied for disinfection. Yari et al. [28] used much higher fortified concentrations (from 10 to 300 mg/L) and reported a maximum depletion of 65.92% after 20 min of direct photolysis using low-pressure UV mercury lamps. The results reported in this study show that medium-pressure lamps that emit polychromatic light are extremely effective for degrading the target neonicotinoid insecticides in real water matrices and should be considered by wastewater and drinking water facilities to ensure the extremely effective photodegradation of these pollutants of concern.

As seen in the literature, the photolytic degradation of caffeine is a very complex process [37,38], with the coupling with other treatment processes [39] being necessary. Specifically, these dissimilar behaviours in the degradation kinetics of caffeine and other organic compounds has been previously described in the literature by Baena-Nogueras et al., who reported a complete photodegradation of many pharmaceutical products and personal care products from complex mixtures, while caffeine was photochemically stable [40]. 

Considering these findings, the next step should be focused on improving the removal rate of caffeine, thus enhancing the overall performance of the process. With this aim, several indirect photolysis assays were planned in order to test the influence of the H_2_O_2_ addition to the solution prior to its treatment.

### 3.2. Removal of a Mixture of Neonicotinoids and Caffeine by Indirect Photolysis

**Figure 4:**
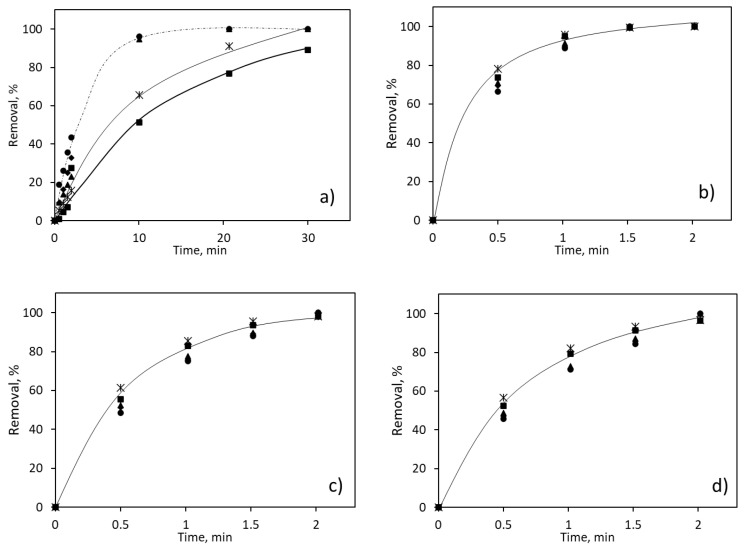
Influence of the H_2_O_2_ addition on the removal of pollutants from the mixture: (**a**) caffeine; (**b**) thiamethoxam; (**c**) clothianidin; (**d**) imidacloprid. H_2_O_2_ concentration: ■—5 mg dm^−3^; *—10 mg dm^−3^; ▲—15 mg dm^−3^; ♦—30 mg dm^−3^; ●—60 mg dm^−3^.

Figure 4 represents the influence of the hydrogen peroxide dosage and irradiation time in the removal of the pollutant mixture. Due to the dissimilar removal efficiency, the time scale used for caffeine (from 0 to 30 min) is different from that employed for neonicotinoids (from 0 to 2 min). Moreover, the data included in Figure 4 were adjusted to pseudo-first-order kinetics, being the time-based direct and indirect photolysis rate constants (K) obtained, described in Table 1.

As can be observed, the complete removal of the insecticides, presented in Figure 4b–d, was achieved in the first two minutes of the assay, regardless of the hydrogen peroxide concentration. However, it is worth mentioning that, in all three cases, the lower removal degrees were obtained with the highest hydrogen peroxide concentration added. This means that an overdosage of H_2_O_2_ will act as a scavenger and will thus interfere in the oxidation reaction [41]. This conclusion can also be obtained from the value of the kinetic constants, which are maximum for a concentration of hydrogen peroxide ranging from 5 to 10 mg dm^−3^. Moreover, it can be stated that the highest kinetic constants are obtained for the degradation of thiamethoxam, for both direct and indirect photolysis. 

Caffeine, on the other hand, presented in the Figure 4a, only achieved the complete removal of concentrations equal to or higher than 15 mg dm^−3^ after 10 min of UV exposure. The indirect photolysis process involves the formation of a hydroxyl radical generated by the rupture of the H_2_O_2_ molecule under UV irradiation. This decomposition by photons occurs with an energy higher than that of the O-O junction, and has an almost-unitary quantum yield (φHO- = 0.98 at 254 nm), producing two HO• for each H_2_O_2_ molecule [42]. This generation of hydroxyl radicals clearly enhances the degradation of caffeine and, thus, improves the performance of the process. According to the kinetic constants obtained, it can be concluded that the optimal concentration of hydrogen peroxide was 15 mg dm^−3^, as further additions of this chemical did not significantly improve the degradation of caffeine.

These results have a direct impact in the electrical energy per order (EEO), calculated in Table 2.

As expected, the power consumption per order of caffeine is the largest, in line with the lower degradation rate measured. The degradation of neonicotinoids is optimal for low concentrations of hydrogen peroxide, being the lowest consumption obtained for the degradation of thiamethoxan by indirect photolysis and a concentration of hydrogen peroxide of 5 mg dm^−3^ (9.5 kWh m^−3^ order^−1^). The power consumption obtained for the degradation of the three neonicotinoids selected was slightly higher than the average reported for this kind of processes, according to the review recently published by Miklos et al. [43]. This higher consumption is justified by the very low and realistic concentration selected for the present work (in the range of ppb), compared to the much-higher concentration usually used in studies that evaluate the degradation kinetics of organic pollutants by photolysis.

### 3.3. Ecotoxicity Analysis

**Figure 5:**
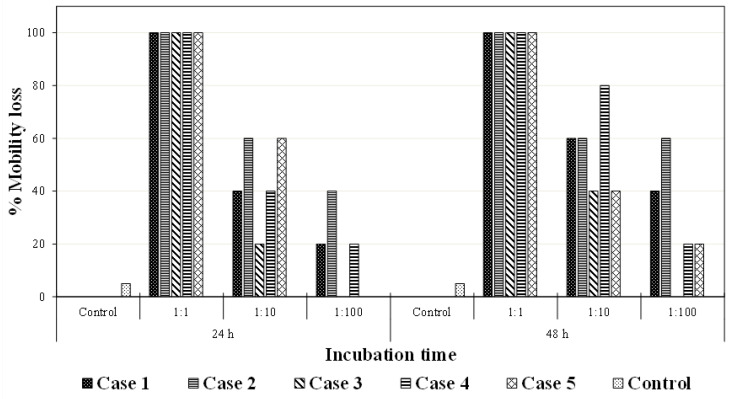
Mobility loss of *Daphnia magna* crustaceans in the ecotoxicity test. Case 1: surface water and bovine liver catalase. Case 2: DP—10 min. Case 3: DP—20 min. Case 4: 15 mg dm^−3^ H_2_O_2_ pollutants—20 min. Case 5: 15 mg dm^−3^ H_2_O_2_—20 min. Control: raw SW.

The performance of the treatment was further evaluated by performing ecotoxicity assays using *Daphnia magna* microcrustaceans as bioindicators. The objective of these tests was to determine the toxicity effect of different samples, evaluating the mobility of *Daphnia magna*. The samples were divided into six cases as follows: surface water, organic pollutants, and bovine liver catalase (Case 1); surface water, organic pollutants, and bovine liver catalase subjected to direct photolysis during 10 min (Case 2) and 20 min (Case 3); surface water, organic pollutants, and bovine liver catalase at 20 min of indirect photolysis and 15 mg dm^−3^ H_2_O_2_ (Case 4); SW and bovine liver catalase at 20 min of indirect photolysis and 15 mg dm^−3^ H_2_O_2_, but without adding the organic pollutants (Case 5); in addition, a sample of raw SW (Negative Control, Case 6) was analyzed in order to evaluate the toxicity of the water matrix. All results are gathered in Figure 5, including exposure to three different dilutions (1:1, 1:10, and 1:100). 

The first observation that can be obtained from these results is that all undiluted samples were toxic enough to induce a mobility loss to all the crustaceans present, regardless of their characteristics. As expected, the mortality began to decrease with the dilution degree, appreciating differences between the samples at 24 and samples at 48 h. Thus, by reducing the concentration 10 times, mobility loss percentages of 60% for cases 2 and 5, and a 40% immobilization in the cases 1 and 4, were observed, while, for case 3, an 80% mobility loss was achieved. By diluting the sample 1:100, a 40% mobility loss for case 2, 20% for cases 1 and 4, and a complete mobility loss for cases 3 and 5 was obtained. On the other hand, by analyzing the results obtained after 48 h, it can be stated that, in the initial sample, the mobility loss was complete. For the dilution 1:10, a 60% mobility loss for cases 1 and 2, 40% for cases 3 and 5, and 80% for case 4 was achieved. 

Several important conclusions can be obtained from comparing the percentage of mobility loss for the different samples. Firstly, an increase in the mobility loss of cases 4 and 5 (both for the 20-min treatment and with hydrogen peroxide) was observed, with respect to case 3 (a 20-min treatment with direct photolysis). The concentration of neonicotinoids was expected to be the same, as 2 min were enough to completely deplete their initial concentration. On the contrary, the concentration of caffeine was expected to be higher for case 3, as the efficiency for the removal of this compound by direct photolysis was much lower than when hydrogen peroxide was added to the treatment. Thus, in this case, the difference in mobility loss could be attributed to the exposure to hydrogen peroxide, which, as seen in the literature, has lethal effects on freshwater crustaceans such as *Daphnia magna* [44].

**Figure 6:**
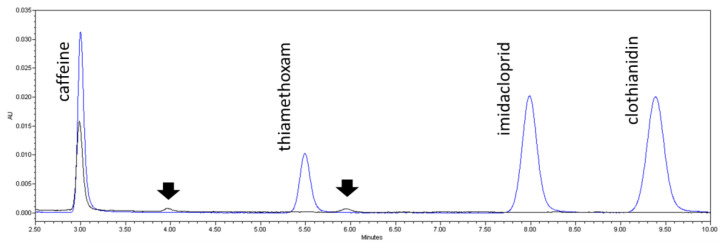
Overlaid chromatograms of samples from photolysis assay at time zero (blue line) and after 10 min of irradiation (black line). Arrows point to the formation of two possible by-products.

It is also worth noting that a higher inhibition was registered for the samples of DP and for 10-min treatments, compared to the mobility loss caused by the initial sample. On the contrary, this mobility loss decreased for 20 min of treatment. As the concentration of the initial pollutants was expected to decrease with time, a plausible explanation would be the formation of by-products. To further explore this assumption, Figure 6 shows the chromatograms of two samples, collected before and after 10 min of photolysis.

The chromatogram obtained before photolysis shows the retention times of each target compound: caffeine appears at minute 3.0, thiamethoxam at minute 5.5, imidacloprid at minute 8.0, and clothianidin at minute 9.4. As can be seen, after photolysis, two small peaks are detected after 10 min of irradiation time (at 3.9 min and 5.9 min of retention time, as shown by the arrows in Figure 6). The latter peak (detected after a run-time of 5.9 min) does not appear after 20 min of direct photolysis. This means that, during direct photolysis, toxic by-products may be formed and depleted by the UV light if further irradiation is applied, resulting in a final solution of lower ecotoxicity than the initial sample. The formation of these by-products and their effect on the toxicity of the samples should be further characterized in future studies.

## 4. Conclusions

The first step of this work consisted in analyzing the decadic molar extinction coefficient of the target pollutants, to check its potential treatment by photolysis using medium-pressure mercury lamps. It was observed that caffeine reaches its maximum absorption at 274 nm, imidacloprid at 272 nm, clothianidin at 246 nm, and thiamethoxam at 251 nm. 

The insecticides are completely removed by direct photolysis in just 2 min of irradiation; meanwhile, caffeine is much more resistant: a 15% removal after 3 h of irradiation time. The caffeine degradation significantly improves with the addition of H_2_O_2_ in the indirect photolysis assay. Moreover, it can be concluded that the optimal concentration of hydrogen peroxide for the complete removal of caffeine is 15 mg dm^−3^; meanwhile, the lowest power consumption regarding the degradation of neonicotinoids is obtained for the lowest range of hydrogen peroxide concentration.

Although further work is needed, the role of the by-products generated was found to be important on the evolution of the toxicity, with direct photolysis being a plausible method for decreasing the concentration of these by-products.

## Figures and Tables

**Table 1:** Values of direct and indirect photolysis rate constants obtained and correlation coefficients (R^2^) for the time-based pseudo-first-order kinetics for the degradation of the target pollutants. DP: Direct Photolysis.

Pollutant	DP		5 mg dm^−3^		10 mg dm^−3^		15 mg dm^−3^		30 mg dm^−3^		60 mg dm^−3^	
	K (min^−1^)	R^2^	K (min^−1^)	R^2^	K (min^−1^)	R^2^	K (min^−1^)	R^2^	K (min^−1^)	R^2^	K (min^−1^)	R^2^
Caffeine	**0.0007**	0.99	**0.07**	0.99	**0.11**	0.99	**0.30**	0.99	**0.33**	0.99	**0.33**	0.99
Thiamethoxam	**2.64**	0.99	**3.38**	0.99	**3.37**	0.99	**2.66**	0.98	**2.25**	0.99	**2.33**	0.99
Clothianidin	**1.68**	0.99	**1.98**	0.99	**2.03**	0.99	**1.48**	0.99	**1.52**	0.99	**1.54**	0.99
Imidacloprid	**1.44**	0.99	**1.68**	0.99	**1.74**	0.99	**1.35**	0.99	**1.33**	0.99	**1.29**	0.99

**Table 2:** Values of the electrical energy per order (EEO, kWh m^−3^ order^−1^) for the degradation of the target pollutants using direct and indirect photolysis. DP: Direct Photolysis.

Pollutant	DP	5 mg dm^−3^	10 mg dm^−3^	15 mg dm^−3^	30 mg dm^−3^	60 mg dm^−3^
Caffeine	45,634.9	456.3	290.4	106.5	96.8	96.8
Thiamethoxam	12.1	9.5	9.5	12.0	14.2	13.7
Clothianidin	19.0	16.1	15.7	21.6	21.0	20.7
Imidacloprid	22.2	19.0	18.4	23.7	24.0	24.8

## Data Availability

Data available in a publicly accessible repository that does not issue DOIs. Publicly available datasets were analyzed in this study. This data can be obtained by contacting the corresponding author of the work at: javier.llanos@uclm.es.

## Sample Availability

Samples of the compounds are not available from the authors.
